# Use of Residual Malt from an Artisanal Beer Brewing Process in the Biosynthesis of Silver Nanoparticles Mediated by Nucleating and Structure-Directing Agents

**DOI:** 10.3390/molecules29071660

**Published:** 2024-04-07

**Authors:** César A. Dueñas-Bolaños, Margarita Cid-Hernández, Gilberto Velázquez-Juárez, Luis A. García-Casillas, Luis J. González-Ortiz, María Judith Sánchez-Peña, Azucena Herrera-González, Oscar Guillermo Zúñiga-González, Edgar J. López-Naranjo

**Affiliations:** CUCEI—Centro Universitario de Ciencias Exactas e Ingenierías, Universidad de Guadalajara, Guadalajara 44430, JAL, Mexico; cesar.duenas7449@alumnos.udg.mx (C.A.D.-B.); margarita.cid@academicos.udg.mx (M.C.-H.); luis.garcia3988@alumnos.udg.mx (L.A.G.-C.); luisj.gonzalezo@academicos.udg.mx (L.J.G.-O.); maria.spena@academicos.udg.mx (M.J.S.-P.); guillermo.zuniga@academicos.udg.mx (O.G.Z.-G.)

**Keywords:** biosynthesis, silver nanoparticles, malt extract, morphological change, green chemistry

## Abstract

Biosynthesized silver nanoparticles (AgNPs) are widely used in varied applications, which are morphology dependent. Consequently, a morphology-controlled synthesis is mandatory. Although there are several studies focused on the plant extract-based biosynthesis of metallic nanoparticles, the use of extracts obtained from agro-wastes is scant. Furthermore, information regarding morphology modification through the use of additional agents is even more scarce. Thus, in this study, AgNPs were synthesized using a malt extract (ME) obtained from an artisanal beer brewing process residue. Additionally, sodium chloride (NaCl), gum arabic (GA), and talc (T) were used in an attempt to modify the morphology of AgNPs. XRD, DLS, SEM, and TEM results demonstrate that stable AgNPs of different sizes and shapes were synthesized. FTIR, HPLC analysis, and the quantification of total proteins, free amino acids, reducing sugars, and total polyphenols before and after AgNPs synthesis showed that ME biomolecules allowed them to act as a source of reducing and stabilizing agents. Therefore, this study provides evidence that ME can be successfully used to biosynthesize AgNPs. Additionally, the antibacterial activity of AgNPs against Gram-negative and Gram-positive bacteria was evaluated. Results indicate that AgNPs show a higher antibacterial activity against Gram-positive bacteria.

## 1. Introduction

Silver nanoparticles (AgNPs) are clusters of silver atoms ranging from 1 to 100 nm in diameter. Among all the noble metal nanoparticles, AgNPs have gained interest because of their unique properties such as chemical stability, good conductivity, and catalytic activity. Furthermore, AgNPs have antibacterial, antifungal, antimicrobial, and antiviral activities. Additionally, evidence has shown that AgNPs have anti-inflammatory effect and accelerate wound healing. These are three main groups of methods to synthesize AgNPs: physical, chemical, and biological. Physical and chemical techniques are the conventional methods used in the AgNPs synthesis; however, both methods are associated with major drawbacks such as the consumption of large amounts of energy (high costs) and the use of toxic substances as reducing agents (e.g., sodium borohydride and hydrazine), which are harmful to the environment. The above fact led to the development of biological methods as an eco-friendly option to synthesize AgNPs [1,2,3,4,5,6,7,8]. AgNPs produced by biological methods show numerous benefits: low cost, easy production at high scales, decrease in energy consumption, and environmental compatibility. Furthermore, biosynthesis does not require the use of elevated temperature, high pressure, or toxic chemical compounds, which implies less risk to human health [2,4,5,6,7,9].

Biological synthesis includes several types of routes. For example, the use of living organisms, such as fungi, plants, algae, yeast, and bacteria that allow the synthesis of AgNPs but imply a certain risk to human health [5,10]. Microorganisms can also be effectively used as biotemplates to fabricate functional AgNPs with controlled morphologies [11]. On the other hand, the use of plants and plant extracts offer a quick, environmentally safe, non-pathogenic, and affordable methodology [1,12,13]. Plant extracts contain biomolecules such as proteins, amino acids, enzymes, polysaccharides, flavonoids, alkaloids, tannins, phenolics, saponins, terpenoids, and vitamins that can be used as stabilizing and reducing agents during the biosynthesis of AgNPs [1,7,14]. Furthermore, evidence has shown that, in contrast to physical and chemical techniques, nanoparticles made from these extracts have stable size and form [15].

Plant extracts can be obtained from various parts of the plant such as leaf, stem, root, bark, fruit, callus, flower, bud, the whole plant [16], or even plant wastes [17]. Table 1 provides information regarding different types of plant extracts and agro-wastes used to biosynthesize AgNPs.

On the other hand, in 2022, 141 million hectoliters of beer was produced in Mexico, making it the fourth largest producer of beer in the world. One of the main ingredients to produce beer is malted barley or malt [32]. Malt is a cereal grain that is treated via a process called malting. This process consists of the germination of the grain by soaking it in water and then stopping the germination by drying it with hot air. Barley is the most selected malted grain because of its high enzyme content, which is related to the germination of this cereal [33,34]. One of the main concerns of this industry is the generation of by-products, with brewer’s spent grain (BSG) being the most abundant as it represents approximately 85% of the total obtained by-products. Additionally, approximately 20 kg of wet BSG is produced per 100 L of brewed beer, and as a consequence, 2.82 million tons of wet BSG was produced in Mexico in 2022 [35,36]. The chemical composition of BSG mainly consists of proteins, lipids, starch, ash, lignin, phenolics, cellulose, hemicellulose, vitamins, and minerals [37]. Since BSG is highly rich in biomolecules and the biomolecules mentioned above can be used as stabilizing and reducing agents, this agro-waste can potentially be used to biosynthesize AgNPs.

In addition, as we can see in Table 1, spherical shape is the most common morphology for biosynthesized AgNPs using plant extracts and agro-wastes. In general, morphology depends on the interaction of the metallic precursor with the reducing and stabilizing agents, as well as on the method of synthesis and the experimental conditions of the process [38]. Morphology modification is of great relevance since it determines the possible applications of AgNPs [39]. To complete this task, the use of nucleating agents (NAs) and structure-directing agents (SDAs) constitutes a viable option.

NAs are compounds that improve the rate of nucleation mechanisms in which solute crystals are not involved in the formation of new crystals (primary nucleation) by reducing the driving force to create a stable nucleus, which results in an increase in the overall crystallization rate of a material [40,41]. On the other hand, SDAs are molecules that facilitate the formation of specified crystal shapes in a shape-selective synthesis by a preferential binding of the SDA on a specific facet of the nanocrystal [42].

**Figure 1:**
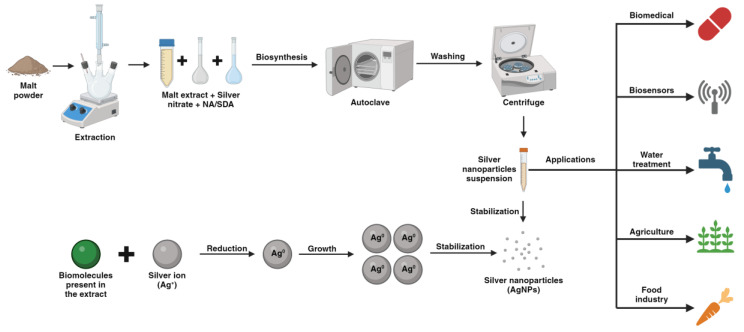
Synthesis process and mechanisms of green synthesis with applications of AgNPs (created with BioRender.com).

Therefore, in this study, AgNPs were biosynthesized using malt extract (ME), obtained from the residues of a beer brewing process, as a source of reducing and stabilizing agents. Additionally, sodium chloride (NaCl) was used as a nucleating agent and gum arabic (GA) or talc (T) as a structure-directing agent to attempt to modify the morphology of the malt extract silver nanoparticles (ME-AgNPs). Figure 1 shows the general synthesis process and mechanism of ME-mediated biosynthesis as well as some possible applications of the AgNPs. Although the mechanistic aspects responsible for biosynthesis of AgNPs are still unknown, it can be said in general that biomolecules reduce metallic ions by means of different functional groups as proposed by Alvarado-Arámburo et al. [43].

## 2. Results

### 2.1. XRD Analysis

**Figure 2:**
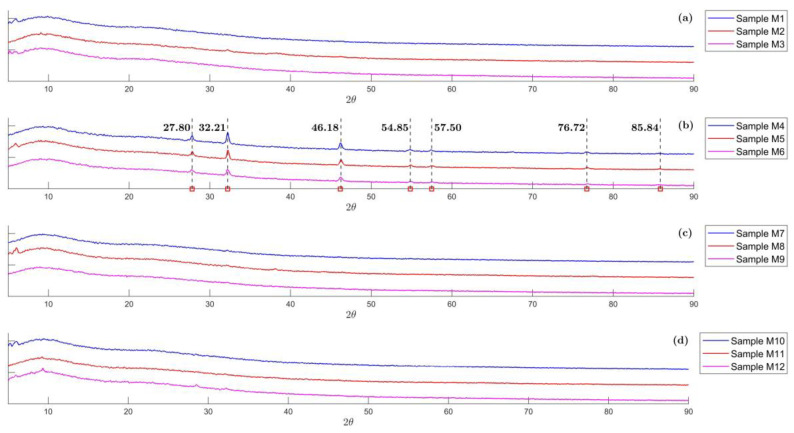
X-ray diffraction (XRD) pattern of the biosynthesized ME-AgNPs: (**a**) No NA/SDA (Samples M1 to M3), (**b**) NaCl (Samples M4 to M6), (**c**) GA (Samples M7 to M9), and (**d**) T (Samples M10 to M12).

The XRD patterns of the biosynthesized ME-AgNPs are shown in Figure 2. The diffractograms of ME-AgNPs with no NA or SDA (Samples M1 to M3), with GA (Samples M7 to M9) and with T (Samples M10 to M12), showed that they were mostly amorphous (Figure 2a,c,d). On the other hand, the diffractograms of ME-AgNPs with NaCl (Samples M4 to M6) showed peaks at 27.80°, 32.21°, 46.18°, 54.85°, 57.50°, 76.72°, and 85.84°, which match up to the (111), (200), (220), (311), (222), (311), and (422) *hkl* planes of the cubic silver chloride, respectively (Figure 2b) [44,45].

Additionally, the relative high intensity of the 27.80° (111), 32.21° (200), and 46.18° (220) peaks in ME-AgNPs with NaCl as an NA suggests that these samples had a preferential growth direction. It also could be stated that as the number of stirring process increased, the crystallinity of the sample decreased since the area under the peaks diminishes from sample M4 to sample M6.

### 2.2. DLS Analysis

The average size and zeta potential of the biosynthesized ME-AgNPs are presented in Table 2. The DLS measurements of the average size confirm the nanometric size of the biosynthesized ME-AgNPs. In addition, the zeta potential data demonstrate that the surface of the nanoparticles is negatively charged and that they are stable in a water suspension. A positive (<30 mV) or negative (>−30 mV) charge on the surface of the AgNPs indicates that nanoparticles are stable and prevents clusters formation [46,47]. According to DLS results, stable nanometric ME-AgNPs were effectively biosynthesized.

The effect of the use of an NA or SDA and the number of stirring cycles was analyzed. The listed values correspond to the means plus or minus the standard deviation (in parenthesis) values of three tests per group. ANOVA tests with *p* value less than 0.05 were performed. *p*-values > 0.2 were obtained. Therefore, no statistical significance was found. Consequently, the number of stirring cycles and the use of NA or SDA does not have significant influence on the average size and the Z-Potential of the biosynthesized ME-AgNPs.

### 2.3. FTIR Analysis

**Figure 3:**
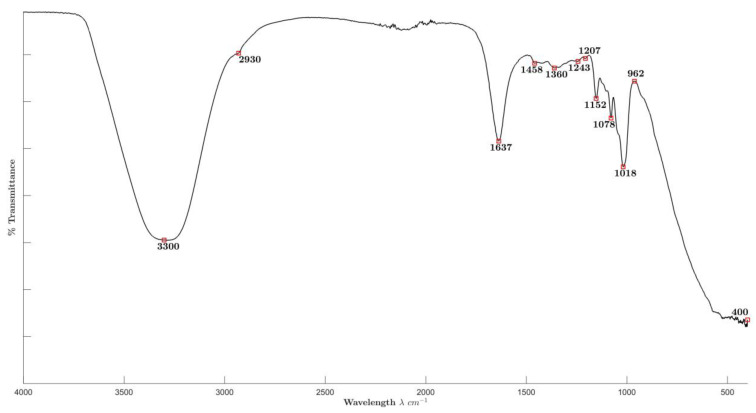
Fourier transform infrared (FTIR) spectra of ME.

Figure 3 shows the FTIR spectra obtained for ME with peaks at 3300, 2930, 1637, 1458, 1360, 1243, 1207, 1152, 1078, and 1018 cm^−1^ and a prominent peak area between 962 and 400 cm^−1^. This spectrum showed several functional groups that could lead to the reduction in the Ag^+^ ions. The 3330 cm^−1^ peak is commonly attributed to O-H stretching in alcohols, phenols, and carboxylic acids and N-H stretching in primary amines. The 2930 cm^−1^ peak corresponds to C-H stretching in alkanes, while the 1637 cm^−1^ peak corresponds to N-H stretching in amines and =C-C= stretching in alkenes. Also, the peak at 1458 cm^−1^ indicates the C-H bending in alkanes, N-O asymmetric stretching in nitroso compounds, and C-C stretching in aromatics (in ring). The peak at 1360 cm^−1^ indicates the C-H rocking in alkanes. On the other hand, the peaks at 1243 and 1207 cm^−1^ correspond to C-N stretching in aliphatic amines. The 1152, 1078, and 1018 cm^−1^ peaks indicate the C-O stretching in alcohols and carboxylic acids. The prominent peak area between 962 and 400 cm^−1^ corresponds to N-H wagging in primary amines and =C-H bending in alkenes. Considering that it has been reported that malt has around 5042 compounds which include amines, alcohols, carboxylic acids, terpenes, etc. [48], and also nitroso compounds such as N-nitrosodimethylamine have been found in malt [49], the detection of the signals of these functional groups on the surface of ME-AgNPs was expected.

**Figure 4:**
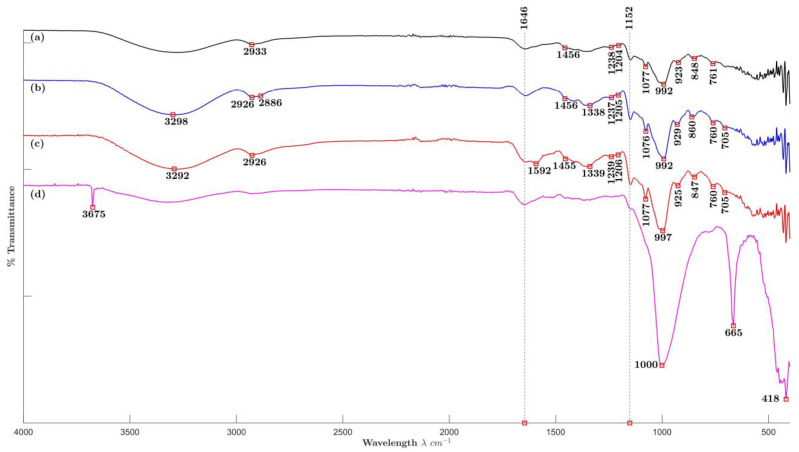
Fourier transform infrared (FTIR) spectra of selected ME-AgNPs: (**a**) No NA/SDA, (**b**) NaCl, (**c**) GA, and (**d**) T.

Figure 4 shows the FTIR spectra of No NA/SDA, NaCl, GA, and T samples of ME-AgNPs (Figure 4a, Figure 4b, Figure 4c and Figure 4d, respectively). Figure 4a peaks correspond to main peaks around 2926, 1646, 1456, 1338, 1240, 1206, 1152, 1077, and 993 cm^−1^ and a prominent peak area between 925 and 400 cm^−1^ previously described for ME (Figure 3), which indicates the presence of ME biomolecules acting as stabilizing agents on the surface of these AgNPs [48,49]. In the case of NaCl/ME-AgNPs (Figure 4b), besides the peaks corresponding to ME, an additional peak at 2886 cm^−1^ corresponding to CH_2_ and CH_3_ stretching is observed. This peak is characteristic of Ag/AgCl-natural extract-based nanoparticles [50]. Regarding GA/ME-AgNPs sample (Figure 4c), additional peak around 1600 cm^−1^ is due to asymmetric and symmetric stretching of COO- group [51]. Finally, T/ME-AgNPs biosynthesized samples (Figure 4d) presented peaks at 1646 and 1152 cm^−1^ previously described for ME. On the other hand, peak located at 3675 cm^−1^ corresponds to O-H stretching in alcohols and phenols, while the prominent peaks at 1000 and 665 cm^−1^ were allocated to Si-O stretching and the peak located at 418 cm^−1^ is related to Si-O-Si bending. These signals confirm the presence of T, which stabilizes the ME-AgNPs without any chemical interaction [52]. The presence of ME functional groups in all samples indicate that the ME biomolecules not only work as the reducing agents but also as the stabilizing agents of the AgNPs.

### 2.4. SEM Analysis

**Figure 5:**
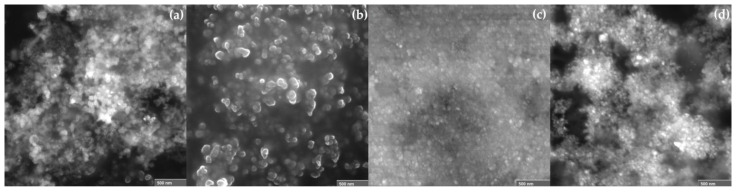
Selected SEM micrographs of the biosynthesized ME-AgNPs: (**a**) No NA/SDA, (**b**) NaCl, (**c**) GA, and (**d**) T.

Selected ME-AgNP micrographs are shown in Figure 5, indicating that different sized-AgNPs were synthesized. Figure 5a shows that without the use of an NA or SDA, quasi-spherical nanoparticles were biosynthesized. On the other hand, Figure 5b shows that with the use of NaCl as an NA, a mixture of morphologies can be observed (i.e., cubes, spheres, and semi-spheres). Figure 5c,d show that irregular-shaped nanoparticles were biosynthesized with GA and T as SDAs.

### 2.5. TEM Analysis

**Figure 6:**
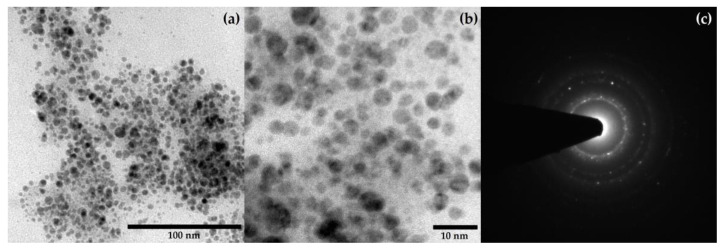
Selected TEM micrographs of the biosynthesized ME-AgNPs (No NA/SDA): (**a**,**b**) Quasi-spherical-shaped AgNPs at different magnifications and (**c**) the SAED pattern of the AgNPs.

Figure 6 shows selected TEM micrographs giving further insights into the biosynthesized AgNPs. The size of AgNPs varied around 4–5 nm with quasi-spherical shape as observed in Figure 6a,b. Additionally, Figure 6c shows the SAED pattern of the AgNPs.

### 2.6. HPLC Analysis of ME before and after AgNP Biosynthesis

The carbohydrate profile of ME was carried out on extract before (ME) and after (ME-AgNPs-S) the biosynthesis of AgNPs. Maltose, glucose, and fructose were identified to be present in the extract. As indicated in Table 3, the content of all sugars reduced after the biosynthesis of ME-AgNPs, confirming their role as reducing and stabilizing agents.

### 2.7. Phytochemical Screening of ME before and after AgNP Biosynthesis

**Figure 7:**
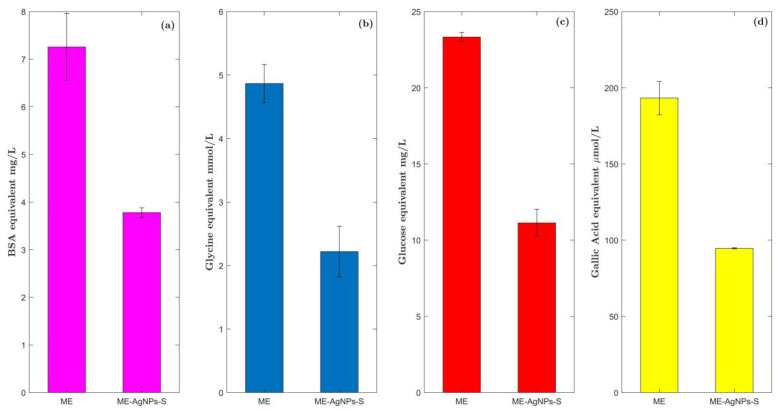
Phytochemical screening (total proteins, amino acids, reducing sugars, and total polyphenols) of the malt extract and the supernatant of ME-AgNPs: (**a**) concentration of total proteins estimated by the bicinchoninic acid method, (**b**) concentration of free amino acids estimated by the ninhydrin method, (**c**) concentration of reducing sugars estimated by the Miller procedure using the dinitrosalicylic acid method, and (**d**) concentration of total polyphenols estimated by the Folin–Ciocalteu method. Values are expressed as mean ± standard deviation (*n* = 3).

Fresh ME and the supernatant of ME-AgNPs (ME-AgNPs-S), which was collected via centrifugation after biosynthesis, were analyzed to quantify metabolite content such as that of amino acids, reducing sugars, proteins, and polyphenols. Total protein concentration was determined by the bicinchoninic acid method using bovine serum albumin equivalent (BSAE) as a reference. ME presented a value of 7.3 ± 0.71 mg BSAE/mL, while a value of 3.8 ± 0.08 mg BSAE/mL were determined for ME-AgNPs-S (Figure 7a). This represents a decrease of 47.9% in total protein concentration compared to ME after the biosynthesis.

Free amino acids were estimated by the ninhydrin method with glycine equivalent (GlyE) as a reference. A concentration of 4.9 ± 0.34 mM was obtained for ME, while a concentration of 2.2 ± 0.38 mM was determined for ME-AgNPs-S. The above indicates that there was a decrease of 54.4% compared to the ME (Figure 7b).

The procedure outlined by Miller was performed using the dinitrosalicylic acid (DNS) method to quantify reducing sugars using glucose equivalents (GluE) as a reference. The ME contained 23.3 ± 0.27 mg GluE/mL. For ME-AgNPs-S, a value of 11.1 ± 0.86 mg GluE/mL was determined, indicating a decrease of 52.3% compared to the ME (Figure 7c).

Finally, total polyphenols were determined using the Folin–Ciocalteu method with gallic acid equivalent (GAE) as a reference. The ME presented a concentration of 193.2 ± 10.95 µM GAE/mL, while a concentration of 94.7 ± 0.40 µM GAE/mL was determined for ME-AgNPs-S, which represents a decrease of 51.0% in polyphenol concentration compared to the ME (Figure 7d).

This decrement in the concentration of these biomolecules may indicate that they act as reducing agents during the biosynthesis of ME-AgNPs.

### 2.8. Antimicrobial Activity

**Figure 8:**
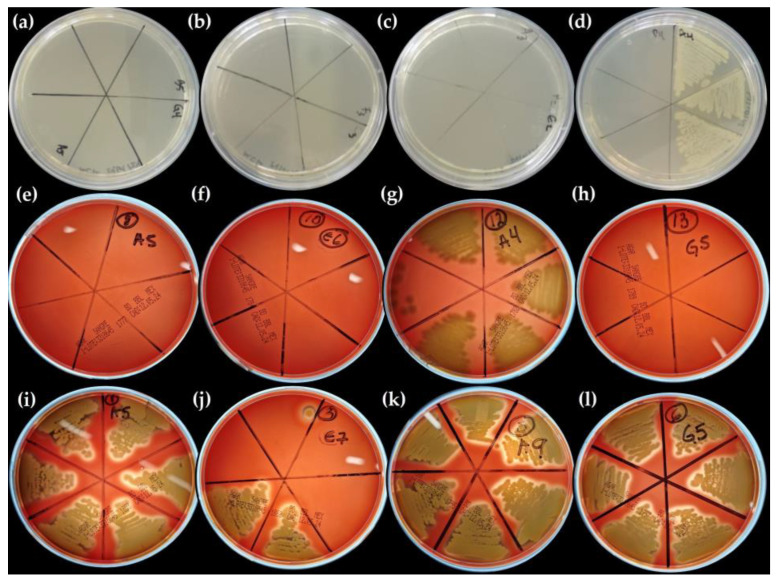
Antibacterial activity of biosynthesized ME-AgNPs [No NA/SDA (M1 and M2), NaCl (M5 and M6), GA (M7 and M8), and T (M11 and M12)] against different Gram-positive and Gram-negative bacteria. *E. coli* [(**a**) (M1 and M2), (**b**) (M5 and M6), (**c**) (M7 and M8), and (**d**) (M11 and M12)], *S. typhimurium* [(**e**) (M1 and M2), (**f**) (M5 and M6), (**g**) (M7 and M8), and (**h**) (M11 and M12)], and *S. aureus* [(**i**) (M1 and M2), (**j**) (M5 and M6), (**k**) (M7 and M8), and (**l**) (M11 and M12)].

Table 4 shows minimal inhibitory concentration for each ME-AgNP sample, while Table 5 shows the colony forming units (CFUs) per Ag amount (mmol), and Figure 8 shows images of the antibacterial tests of AgNPs against *E. coli* (Figure 8a–d), *S. typhimurium* (Figure 8e–h), and *S. aureus* (Figure 8i–l). According to the results, AgNPs biosynthesized using NaCl (Figure 8b,f,j) as an NA showed the highest antibacterial effect since lower [mM Ag] was required to inhibit the tested bacterial strains. The use of NaCl during the synthesis of AgNPs could have interacted with Ag+ ions to form AgCl, and consequently, its presence increased the bacteriostatic properties of these samples [53,54,55]. Regarding the use of GA (Figure 8c,g,k) and T (Figure 8d,h,l), it seems that their use had a deleterious effect since these samples showed a lower antibacterial effect than samples synthesized without the use of additional agents. Finally, it seems that AgNPs showed a higher antibacterial effect against *S.aureus* (Gram-positive bacteria) than *E. coli* or *S. typhimurium* (Gram-negative bacteria) since a lower Ag concentration was required to kill *S. aureus*. Although a great number of publications indicate that Gram-negative bacteria are generally more susceptible to AgNPs compared to Gram-positive bacteria, some studies suggest that AgNPs derived from natural sources are more effective against Gram-positive bacteria [56]. For example, Awwad et al. (2020) found larger zones of inhibition for Gram-positive bacteria (*S.aureus*) [57], and Lee et al. (2016) also determined larger zones of inhibition for *B.cereus* (Gram-positive) than for *P. aeruginosa* (Gram-negative) with AgNPs [58].

## 3. Materials and Methods

### 3.1. Materials

AgNO_3_ (99.9999%, Sigma-Aldrich, St. Louis, MO, USA), NaCl (99.5%, Karal, León, Mexico), gum arabic (Sigma-Aldrich, St. Louis, MO, USA), talc powder (Sigma-Aldrich, St. Louis, MO, USA), and ethanol (Almacén de Drogas La Paz, Guadalajara, Jal, Mexico) were used as received. Malt powder was donated by Centro de Investigación y Asistencia en Tecnología y Diseño del Estado de Jalisco A.C. (CIATEJ).

### 3.2. Preparation of ME

To obtain ME, 10 g of malt powder was weighed and added to a 3-neck flask with reflux using 100 mL of distilled water previously boiled at 100 °C. The system was then kept at 100 °C for 30 min. Subsequently, the extract was centrifuged at 4000 rpm at room temperature for 7 min to remove solid wastes. ME was stored in a freezer at 0 °C.

### 3.3. Biosynthesis of AgNPs

To biosynthesize ME-AgNPs, an aqueous solution of 10 mM AgNO_3_ was prepared using 85.20 mg of AgNO_3_ in a 100 mL volumetric flask with distilled water. Additional agents (i.e., NaCl, GA, and T) and modifications in the stirring process during the synthesis of AgNPs were analyzed. A total of twelve samples were synthesized using an autoclave according to the experimental conditions described in Table 6. Once the biosynthesis was performed, ME-AgNPs were centrifugated at 4000 rpm for 7 min and washed three times with ethanol and distilled water.

### 3.4. Characterization of ME-AgNPs

#### 3.4.1. X-ray Diffraction (XRD) Analysis

ME-AgNPs were analyzed to identify their crystalline structure by XRD using a Panalytical Empyream device with Cu, λ = 1.54059 Å, operating at 40 kV and 30 mA, with a scanning range (*2θ*) from 5 to 90 degrees and a step of 0.026 degrees.

#### 3.4.2. Dynamic Light Scattering (DLS)

The average size and zeta potential of ME-AGNPs were obtained using Malvern Zetasizer Nanoseries Nano-ZS90 device. Analysis was performed at 25 °C with 15 scans in triplicate with 15 s of scanning time for each sample. ME-AgNPs were sonicated for 30 mi and dispersed prior to testing using a Vortex Genie 2 (Scientific Industries, Bohemia, NY, USA).

##### Experimental Design and Statistical Analysis

A two factorial experimental design was used to analyze DLS results, where the two factors considered were the number of stirring processes (zero, one, and two) and the use of an NA or SDA (No NA or SDA, NaCl, GA, and T). This procedure was executed using a two-way analysis of variance (ANOVA) (*p* ≤ 0.05) using Statgraphics Centurion 19.

#### 3.4.3. Fourier Transform Infrared (FTIR) Spectroscopy Analysis

ME and ME-AgNPs were analyzed to identify the functional groups present on the surface of ME-AgNPs. The spectrum data were obtained using a Nicolet S50 FTIR device with a one-reflection horizontal attenuated total reflectance (ATR) accessory, where 20 scans were recorded for each sample from 4000 to 400 cm^−1^.

#### 3.4.4. Scanning Electron Microscopy (SEM) Analysis

Scanning electron microscopy was performed using a TESCAN MIRA-3 LMU, operating at 20 kV, to examine the size and shape of the biosynthesized ME-AgNPs.

#### 3.4.5. Transmission Electron Microscopy (TEM) Analysis

Transmission electron microscopy was performed using a JEOL 200-CX operating at 100 kV to examine the size and shape of the biosynthesized ME-AgNPs. AgNPs were deposited on an FF 300 square mesh copper grid for observation.

#### 3.4.6. High-Performance Liquid Chromatography (HPLC)

The quantification of maltose, glucose, and fructose was carried out using HPLC-RI on a Waters Acquity HPLC system (Milford, MA, USA) chromatograph equipped with a Biorad Carbohydrate Analysis column (Aminex HPX-87 C 250 × 4.0 mm, Hercules, CA, USA) and a Acquity Refractive Index Detector. The mobile phase was ultrapure water (18 MΩ cm), the flow rate was 0.30 mL min^−1^, and the temperature of the oven was 65 °C. Maltose, glucose, and fructose were used as standards. The samples were diluted in ultrapure water and filtered through a 0.45 μm nylon syringe filter. Data acquisition and processing were performed with the Empower 3 Chromatography data system software.

### 3.5. Phytochemical Screening of ME before and after the Reaction

#### 3.5.1. Supernatant Obtention

After the biosynthesis of ME-AgNPs, samples were centrifuged at 4000 rpm and room temperature for 7 min. Supernatant was separated from the nanoparticle pellets and stored at 4 °C for future analysis.

#### 3.5.2. Total Protein Concentration

The bicinchoninic acid method was employed to ascertain the total protein concentration. The Pierce™ BCA Protein Assay Kit from ThermoFisher™ was utilized, adhering to the manufacturer’s guidelines for the microplate procedure. Protein concentrations were determined using a calibration curve based on bovine serum albumin (BSA) ranging from 0 to 250 µg/mL. The results were expressed as BSA equivalents (µg) per milliliter of the sample.

#### 3.5.3. Free Amino Acid Estimation

The free amino acid content was estimated using the general procedure for determining amino acids using Product No. N 7285 Ninhydrin Reagent (Sigma-Aldrich), with some modifications [59]. For this purpose, 200 µL of sample and 100 µL of ninhydrin reagent were placed in a microtube; then, the reaction mixture was heated at 90 °C for 3 min. Subsequently, 50 µL of the reaction mixture was diluted with 150 µL of ethanol in a 96-well microplate, and the optical density was measured at 570 nm. A glycine calibration curve ranging from 0 to 2.5 mmol/L was employed, and the results were expressed as glycine equivalents (mmol/L).

#### 3.5.4. Reducing Sugars

Reducing sugars were quantified using the dinitrosalicylic acid (DNS) method, following the procedures outlined by Miller [60]. A glucose calibration curve of 0 to 1.0 mg/mL was established. A 1% solution of DNS served as the working reagent. A 250 µL volume of sample or calibration point was mixed with an equal 1% DNS reagent volume and heated at 99 °C for 5 min. Following cooling, 15 µL of each reaction mixture was transferred to a 96-well microplate and diluted with 185 µL of water before measuring the optical density at 575 nm. The results were expressed as glucose equivalents (mg) per milliliter of the sample.

#### 3.5.5. Total Phenolic Content

The total phenolic content was determined using the Folin–Ciocalteu method [61]. A 20 µL volume of sample was combined with 100 µL of Folin–Ciocalteu reagent in a 96-well microplate. Subsequently, after 60 s, 75 µL of Na_2_CO_3_ solution (10% *w*/*v*) was added. Following an incubation period of 120 min in darkness, the optical density was measured at 760 nm. The results were calculated utilizing a gallic acid calibration curve ranging from 0 to 100 µg/mL and were expressed as gallic acid equivalents (µg) per milliliter of sample.

All measurements were obtained using a UV-vis spectra Thermo Scientific™ Multiskan SkyHigh Microplate Spectrophotometer (Thermo Fisher Scientific, Waltham, MA, USA) and were performed in triplicate.

### 3.6. Antibacterial Activity

A total of three microorganisms were utilized to test the antibacterial properties of the ME-AgNPs and the ME. Gram-negative bacterial strains were *Escherichia Coli* (ATCC 25922), which was used as a control. *Salmonella typhimurium* (ATCC 14028) and *Staphylococcus aureus* (ATCC 29123) were employed as Gram-negative and Gram-positive bacterial strains, respectively. Before the determination of minimal inhibitory concentration, these strains were inoculated onto tryptic soy agar (TSA) and incubated for 24 h at 37 °C.

The bacterial strains were donated by Laboratorio de Inocuidad de los Alimentos, and the assays were performed at Laboratorio de Biotecnología Alimentaria, both located at Centro Universitario de Ciencias Biológicas y Agropecuarias (CUCBA), Universidad de Guadalajara, 44600 Zapopan, Jal, Mexico.

#### Determination of Minimal Inhibitory Concentration (MIC)

The antibacterial activity of the ME-AgNPs and the ME was evaluated by the determination of the minimal inhibitory concentration (MIC) in conformity with the Clinical and Laboratory Standard Institute protocols [62]. For this assay, it was required that the turbidities of bacterial inocula were adjusted to a scale of 0.5 of the McFarland standard, which is equivalent to a cell density of 10^8^ CFU/mL. Afterwards, a serial of dilutions was performed to obtain a concentration of 10^5^ CFU/mL. Additionally, 60 µL of the antibiotic Ampicillin (1.280 µg/mL) was used as a negative control. Moreover, a positive control consisting of 10 µL of each bacterial culture was used separately.

The MIC of each sample was determined by the broth microdilution method. This assay was carried out in 96-well microtiter plates. A 100 µL volume of Mueller Hinton Broth (MHB) was added from the first to the twelfth well of the 96-well microtiter plates. Afterwards, 100 µL of the ME-AgNPs and the ME were added to the first well, separately. Next, the content of the first well was homogenized, and 100 µL of this well was transferred to the second well. This procedure was repeated until the twelfth well. Then, 10 µL of the bacterial suspension was added to all the wells. The concentration of each bacterial solution was of 5.21 × 10^4^ CFU/mL, 1.19 × 10^4^ CFU/mL, and 5.74 × 10^4^ CFU/mL for *Escherichia coli, Salmonella typhimurium, and Staphylococcus aureus*, respectively. This procedure was performed in triplicate for the ME-AgNPs and the ME. The same protocol was followed to assay the antibiotic Ampicillin. All 96-well microtiter plates were incubated for 24 h at 37 °C and were read in a UV-vis spectrophotometry (Agilent Biotek Epoch 2, Agilent, Santa Clara, CA, USA) instrument at a wavelength of 620 nm. The microdilutions that presented the lower optical density (OD_620 nm_) were considered as the alleged MICs.

In order to confirm the MIC, a sample of each microdilution with the lowest OD_620 nm_ was spread and inoculated in Tryptone Soy Agar (TSA) (for *E. coli*) and Blood Agar (BA) (*S. typhimurium and S. aureus*) and incubated for 24 h at 37 °C. The microdilutions that did not present bacterial growth were considered as the MICs of the corresponding evaluated sample. The samples that presented bacterial growth were considered as false positives, and the earlier microdilutions were considered as the MICs.

## 4. Conclusions

This study presented a straightforward and environmentally friendly method of modifying the size and shape of AgNPs using non-toxic compounds and an agro-waste as sources of reducing and stabilizing agents. XRD results demonstrated that the samples synthesized without additional agents or with an SDA were mostly amorphous, while the samples obtained using NaCl showed a crystalline structure. DLS results confirmed the nanometric size and stability of the biosynthesized ME-AgNPs; furthermore, the statistical analysis of the DLS results demonstrates that the number of stirring cycles and the use of an NA or SDA does not have significant influence on the average size and the zeta potential of the nanoparticles. FTIR study confirmed that the functional groups present in ME were transferred to the surface of ME-AgNPs. SEM micrographs showed the different morphologies that were obtained. Phytochemical analyses show that the biomolecule content decreased after the biosynthesis; thus, it may be concluded that these biomolecules acted as reducing agents during the biosynthesis of AgNPs. Finally, AgNPs showed a higher inhibition effect on Gram-positive bacteria.

## Figures and Tables

**Table 1:** AgNPs biosynthesized using plant extracts and agro-wastes.

Source of the Extract	Involved Plant/Agro-Waste	Size (nm)	Shape	Reference
Plant’s leaf	*Tectona grandis*	28	Spherical	[18]
	*Tephrosia purpurea*	~100	Spherical	[19]
	*Moringa oleifera*	25.235 ± 0.694	Spherical	[20]
Plant’s peel	*Citrus limon*	32	Spherical	[21]
	*Punica granatum*	28	Spherical	[21]
	*Hylocereus* spp.	7	Spherical	[22]
Plant’s stem	*Commiphora gileadensis*	13	Spherical	[23]
	*Indigofera macrophylla*	18.09 ± 4.13	Spherical	[24]
	*Scutellaria multicaulis*	~60	Spherical and oval	[25]
Plant’s fruit	*Cucumis sativus var. hardwickii*	11.12–39	Spherical	[26]
	*Olea europaea*	77	Spherical	[27]
	*Artocarpus lakoocha*	6.59–25	Spherical	[28]
Agro-waste	*Cicer arietinum* (black chickpea peels)	47.46	Spherical	[29]
	*Theobroma cacao* (cocoa pod shells)	48.83–55.24	Spherical	[30]
	*Carya illinoinensis* (pecan nutshell)	15–35	Spherical	[31]

**Table 2:** DLS measurements.

Sample	Utilized NA/SDA	DLS	
		Average Size [nm]	Z-Potential (mV)
M1	-	4.733 ± 1.128	−8.84 ± 0.23
M2	-	4.104 ± 0.475	−8.95 ± 1.34
M3	-	3.899 ± 0.712	−8.42 ± 0.38
M4	NaCl	4.209 ± 0.379	−14.07 ± 0.55
M5	NaCl	3.347 ± 0.045	−17.27 ± 0.50
M6	NaCl	3.945 ± 0.456	−8.79 ± 0.69
M7	GA	3.791 ± 0.749	−11.56 ± 1.99
M8	GA	4.613 ± 1.181	−17.33 ± 1.30
M9	GA	4.286 ± 0.738	−8.65 ± 0.42
M10	T	4.174 ± 0.548	−10.33 ± 0.35
M11	T	3.601 ± 0.107	−9.47 ± 0.73
M12	T	4.300 ± 1.092	−11.47 ± 0.32

**Table 3:** HPLC-based carbohydrate profile quantification.

Sample	Maltose gL^−1^	Glucose gL^−1^	Fructose gL^−1^
ME	20.40 ± 0.79	2.4 ± 0.14	0.57 ± 0.07
ME-AgNPs-S	8.32 ± 0.41	1.47 ± 0.34	0.44 ± 0.09

**Table 4:** Antibacterial activity of ME-AgNPs.

**Bacterial Strain**	**Minimal Inhibitory Concentration of the Samples [mM Ag]**					
	**No NA/SDA**			**NaCl**		
	**M1**	**M2**	**M3**	**M4**	**M5**	**M6**
*Escherichia coli*	0.1875	0.1875	0.1875	0.1875	0.09375	0.1875
*Salmonella typhimurium*	0.1875	0.1875	0.1875	0.1875	0.09375	0.09375
*Staphylococcus aureus*	0.375	0.1875	0.1875	0.09375	0.046875	0.09375
**Bacterial Strain**	**Minimal Inhibitory Concentration of the Samples [mM Ag]**					
	**GA**			**T**		
	**M7**	**M8**	**M9**	**M10**	**M11**	**M12**
*Escherichia coli*	0.375	0.375	1.5	0.75	0.75	0.375
*Salmonella typhimurium*	0.375	0.375	0.75	0.1875	0.1875	0.09375
*Staphylococcus aureus*	0.375	0.375	0.75	0.1875	0.375	0.1875

**Table 5:** Quantified inhibition of each bacterium per sample.

Group	Sample	Former Colony Units Inhibited by Millimole of Silver [CFU/mmol Ag]		
		*Escherichia coli*	*Salmonella typhimurium*	*Staphylococcus aureus*
No NA/SDA	M1	2.78 × 10^8^ ± 9.51 × 10^7^	6.35 × 10^7^ ± 5.06 × 10^6^	1.53 × 10^8^ ± 1.58 × 10^7^
	M2	2.78 × 10^8^ ± 9.51 × 10^7^	6.35 × 10^7^ ± 5.06 × 10^6^	3.06 × 10^8^ ± 3.15 × 10^7^
	M3	2.78 × 10^8^ ± 9.51 × 10^7^	6.35 × 10^7^ ± 5.06 × 10^6^	3.06 × 10^8^ ± 3.15 × 10^7^
NaCl	M4	2.78 × 10^8^ ± 9.51 × 10^7^	6.35 × 10^7^ ± 5.06 × 10^6^	6.12 × 10^8^ ± 6.30 × 10^7^
	M5	5.56 × 10^8^ ± 1.90 × 10^8^	1.27 × 10^8^ ± 1.01 × 10^7^	1.22 × 10^9^ ± 1.26 × 10^8^
	M6	2.78 × 10^8^ ± 9.51 × 10^7^	1.27 × 10^8^ ± 1.01 × 10^7^	6.12 × 10^8^ ± 6.30 × 10^7^
GA	M7	1.39 × 10^8^ ± 4.75 × 10^7^	3.17 × 10^7^ ± 2.53 × 10^6^	1.53 × 10^8^ ± 1.58 × 10^7^
	M8	1.39 × 10^8^ ± 4.75 × 10^7^	3.17 × 10^7^ ± 2.53 × 10^6^	1.53 × 10^8^ ± 1.58 × 10^7^
	M9	3.47 × 10^7^ ± 1.19 × 10^7^	1.59 × 10^7^ ± 1.27 × 10^6^	7.65 × 10^7^ ± 7.88 × 10^6^
T	M10	6.95 × 10^7^ ± 2.38 × 10^7^	6.35 × 10^7^ ± 5.06 × 10^6^	3.06 × 10^8^ ± 3.15 × 10^7^
	M11	6.95 × 10^7^ ± 2.38 × 10^7^	6.35 × 10^7^ ± 5.06 × 10^6^	1.53 × 10^8^ ± 1.58 × 10^7^
	M12	1.39 × 10^8^ ± 4.75 × 10^7^	1.27 × 10^8^ ± 1.01 × 10^7^	3.06 × 10^8^ ± 3.15 × 10^7^

This assay was performed in triplicate, and values are expressed as mean ± standard deviation (*n* = 3).

**Table 6:** Conditions of biosynthesis.

Sample	AgNO_3_ (mL of 10 mM Solution)	First Stirring (min)	ME (mL)	Reaction Time (min)	Temperature of Reaction (°C)	Second Stirring (min)	NA or SDA (mL of 10 mM Solution)
M1	3	-	3	15	120	-	-
M2	3	15	3	15	120	-	-
M3	3	15	3	15	120	15	-
M4	3	-	3	15	120	-	NaCl
M5	3	15	3	15	120	-	NaCl
M6	3	15	3	15	120	15	NaCl
M7	3	-	3	15	120	-	GA
M8	3	15	3	15	120	-	GA
M9	3	15	3	15	120	15	GA
M10	3	-	3	15	120	-	T
M11	3	15	3	15	120	-	T
M12	3	15	3	15	120	15	T

## Data Availability

Data are contained within the article.
